# Grasping the semantic of actions: a combined behavioral and MEG study

**DOI:** 10.3389/fnhum.2022.1008995

**Published:** 2022-12-13

**Authors:** Elisa Visani, Gioacchino Garofalo, Davide Rossi Sebastiano, Dunja Duran, Laila Craighero, Lucia Riggio, Giovanni Buccino

**Affiliations:** ^1^Bioengineering Unit, Fondazione IRCCS Istituto Neurologico Carlo Besta, Milan, Italy; ^2^Division of Neuroscience, Universitity “Vita-Salute” San Raffaele, Milan, Italy; ^3^IRCCS San Raffaele, Milan, Italy; ^4^Neurophysiology Unit, Fondazione IRCCS Istituto Neurologico Carlo Besta, Milan, Italy; ^5^Department of Neuroscience and Rehabilitation, University of Ferrara, Ferrara, Italy; ^6^Unit of Neuroscience, Department of Medicine and Surgery, University of Parma, Parma, Italy

**Keywords:** semantics, embodiment, language processing, motor responses, beta rhythm, MEG (magnetoencephalography), sensorimotor system

## Abstract

There is experimental evidence that the brain systems involved in action execution also play a role in action observation and understanding. Recently, it has been suggested that the sensorimotor system is also involved in language processing. Supporting results are slower response times and weaker motor-related MEG Beta band power suppression in semantic decision tasks on single action verbs labels when the stimulus and the motor response involve the same effector. Attenuated power suppression indicates decreased cortical excitability and consequent decreased readiness to act. The embodied approach forwards that the simultaneous involvement of the sensorimotor system in the processing of the linguistic content and in the planning of the response determines this language-motor interference effect. Here, in a combined behavioral and MEG study we investigated to what extent the processing of actions visually presented (i.e., pictures of actions) and verbally described (i.e., verbs in written words) share common neural mechanisms. The findings demonstrated that, whether an action is experienced visually or verbally, its processing engages the sensorimotor system in a comparable way. These results provide further support to the embodied view of semantic processing, suggesting that this process is independent from the modality of presentation of the stimulus, including language.

## Introduction

Accumulating evidence suggests that during action perception the same neural structures necessary for the execution of that action are recruited (Jeannerod et al., 1995; Rizzolatti et al., 1998; Fogassi et al., 2001; Rizzolatti and Matelli, 2003; Buccino et al., 2004; Binkofski and Buccino, 2006; Hardwick et al., 2018; Borra and Luppino, 2019). A matching mechanism in which the visual processing of an action activates a corresponding motor representation has been forwarded, namely action re-enactment, in order to attribute meaning and decoding others’ behavior (Gallese et al., 1996; Rizzolatti and Craighero, 2004; Rizzolatti and Fogassi, 2014). At a neuronal level, this matching mechanism has its counterpart in the presence of the so-called mirror neurons (Di Pellegrino et al., 1992). In the monkey, these neurons were recorded in the ventral premotor area F5 and in the inferior parietal area PFG, and constitute a fronto-parietal network devoted to planning actions and understanding others’ motor acts (Rizzolatti and Fogassi, 2014).

In humans, the neural structures implicated in the execution and understanding of observed actions also appear involved in the understanding and processing of action-related language (Vigliocco et al., 2011; Marino et al., 2014; Borghi and Riggio, 2015; Buccino et al., 2016, 2018; García and Ibáñez, 2016; Sakreida et al., 2016; Zhang et al., 2016; Horoufchin et al., 2018; Garofalo et al., 2022; Visani et al., 2022). In this respect, accumulating empirical evidence suggested that the sensorimotor and even emotional systems involved in experiencing the content expressed by verbal material are causally involved in understanding the content of that linguistic stuff (Pulvermüller, 2002, 2010; Barsalou, 2008; Fischer and Zwaan, 2008; Gallese, 2008; Jirak et al., 2010; Buccino et al., 2016).

In addition, there is evidence that depending on the effector typically used in the expressed action, language that describes that action recruits the sector of the motor system where the effector is motorically represented (Hauk et al., 2004; Tettamanti et al., 2005; Aziz-Zadeh et al., 2006; Baumgaertner et al., 2007; Kemmerer et al., 2008; de Vega et al., 2014). In keeping with this, studies using neurophysiological techniques demonstrated that when processing verbs connected to concrete actions, the motor system is recruited quite early, just 150–170 ms after verbal stimuli are presented auditorily or visually (Pulvermüller et al., 2001; Pulvermüller and Shtyrov, 2005; Pulvermüller et al., 2005). The behavioral counterpart of this early activation is an interference effect. In detail, when participants are required to solve a hand-related semantic task and to give a motor response with the same effector, there is a slowing down of reaction times (Buccino et al., 2005; Boulenger et al., 2006; Sato et al., 2008; Dalla Volta et al., 2009; de Vega et al., 2013, 2014). This interference effect has been explained in terms of competition of neuronal resources (Buccino et al., 2005; de Vega et al., 2013). Specifically, in this context, the motor system is simultaneously processing the meaning of the action as well as preparing the motor response needed to complete the task, leading to a cost for the motor system that will be less prompt to give the motor output. In support of this interpretation, Transcranial Magnetic Stimulation (TMS) studies showed a decrease in Motor-Evoked Potentials (MEPs) during verb listening (Buccino et al., 2005). Furthermore, a weaker suppression of Beta band oscillations was found when motor responses are given with the same body part normally used to perform the action expressed by the verb (Klepp et al., 2015; Visani et al., 2022). Beta band oscillations are the main rhythm deriving from the motor cortex, and it is characterized by a pattern of suppression and rebound during movement (Pfurtscheller and Lopes da Silva, 1999). Beta suppression, or event-related desynchronization (ERD), starts several 100 ms before the start of the movement (both when the movement is internally or externally triggered) and becomes maximal around the time of movement. Hence, due to these features, the ERD is frequently used to investigate the brain correlates of action-related processes, such as action observation (Hari and Kujala, 2009; Moreno and de Vega, 2013), motor imagery (Schnitzler et al., 1997; de Lange and Roelofs, 2008; Brinkman et al., 2014), and the processing of action-related language (Weiss and Mueller, 2012; Klepp et al., 2015; Visani et al., 2022). In general terms, a weaker suppression (minor decrease of ERD) of Beta rhythm indicates that the motor system is less ready for generating a motor output, while an increased suppression (greater decrease of ERD) indicates that the motor system is more ready to generate a motor response.

A still open question is related to the degree of overlap among action execution, understanding of actions visually presented, and understanding of actions verbally described. In a very recent behavioral study of our group (Garofalo et al., 2022), the interference effect was found during the processing of visually presented actions (i.e., pictures depicting hand- and foot-related actions) and actions verbally described (i.e., verbs expressing hand- and foot-related actions). The results of a go/no-go task revealed that when hand actions and hand-related verbs were presented, hand motor responses were slower than when foot actions and foot-related-verbs were presented. We hypothesized that the same semantic mechanisms underlie the understanding of observed actions and verbs.

The aim of the current study was to investigate the neurophysiological underpinnings of the interference effect using oscillatory Magnetoencephalography (MEG) analysis to study Beta band power suppression. The MEG signals were recorded while the participants carried out the same go/no-go task used in a previous study (Garofalo et al., 2022). We expected a replica of the behavioral results and, according to the hypothesis of competition of neuronal resources (Buccino et al., 2005; de Vega et al., 2013), a weaker ERD in both visual and verbal presentation of hand-related actions and verbs as compared to foot-related actions and verbs.

## Materials and Methods

### Participants

Fifteen volunteers (eight females, age = 26.8 ± 5.1 years) were recruited for the experiment. All participants were adult (>18 years), right-handed, according to the Edinburgh Handedness Inventory (Oldfield, 1971), had a normal or corrected-to-normal vision, and were native Italian speakers. Exclusion criteria were formal education in linguistics, the presence of neurological or psychiatric disorders, and the current use of drugs affecting the central nervous system. The experiment was carried out in accordance with the ethical standards laid down in the 1964 Declaration of Helsinki and its later amendments. The experiment was approved by the local Ethical Committee (approval number: 47/2012; date of approval: November 2012). Participants gave their written informed consent before being included in the study.

### Task

**Figure 1:**
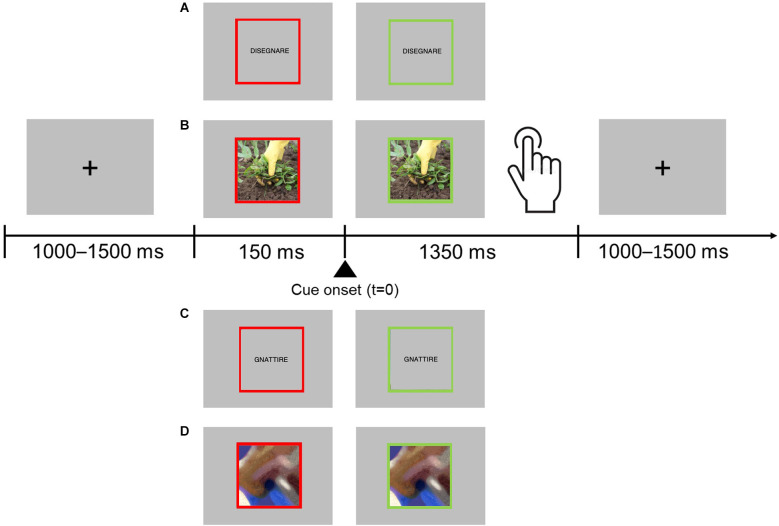
Experimental procedure. Participants were asked to fixate on the center of the screen placed in front of them. Each trial started with the presentation of the stimulus surrounded by a red frame. The stimulus could be either a hand or foot verb, a hand or foot action image, and a pseudoverb or a scrambled image. After 150 ms the frame turned green, and the participants were allowed to respond. Participants were instructed to respond only to words and pictures depicting hand- or foot-related actions. The trial ended when participants provided their responses or after 1,350 ms if no response was given. Stimuli examples: hand action verb **(A)**, hand action image **(B)**, pseudoverb **(C)**, scrambled image **(D)**.

Participants in the experiment were required to perform a go/no-go task in which they had to respond to words and pictures that represented actions involving either hands or feet, and to refrain from responding when presented stimuli were pseudowords (i.e., built by substituting one consonant and one vowel in two distinct syllables of each verb) or scrambled images (see also, Garofalo et al., 2022), during MEG signals acquisition. Participants had to respond with a flexion of the hand and reaction times (RTs) were collected. Each trial started with a black fixation cross displayed at the center of a gray background. After a random delay ranging from 1,000 to 1,500 ms (in order to avoid response habituation), the fixation cross was replaced by a stimulus item, either a hand or foot verb, or pseudoverbs, or a hand or foot image, or a scrambled image. Stimuli were centrally displayed and surrounded by a red frame. The red frame changed to green 150 ms after the stimulus onset. The “go” signal for the response was the change in the frame color. Participants were instructed to give the motor response (hand flexion). After the go signal, stimuli remained visible for 1,350 ms or until participants’ responses (see Figure 1). The experimental task was divided into two sessions, and each part included 48 go trials (12 hand action images, 12 foot action images, 12 hand action verbs, 12 foot action verbs) and 48 no-go trials (12 hand and 12 foot action pseudo-verbs, 12 hand and 12 foot action scrambled images). The stimuli selection procedure as well as their description are illustrated in previous articles of our group (Garofalo et al., 2022; Visani et al., 2022). Stimuli were randomly presented. No feedback was given to participants. Stimuli were delivered using the software package Stim2. Before starting the acquisition, participants underwent a short training session.

### MEG acquisition and pre-processing

A 306-channel whole head MEG system (Triux, MEGIN, Helsinki, Finland) was used to collect the MEG signals. Pairs of electrodes positioned bilaterally 2–3 cm apart over the belly of the right and left flexor and extensor of the wrist were used to simultaneously record surface EMG signals. Signals were sampled at 1 kHz. Moreover, bipolar electro-oculographic (EOG) and electrocardiographic signals (ECG) were acquired. Five head position identification (HPI) coils on the participant’s scalp continuously monitored the participant’s head position inside the MEG helmet. A 3D digitizer (FASTRAK, Polhemus, Colchester, VT, USA) was used to digitally capture the locations of these coils, three anatomical landmarks (the nasion, right and left preauriculars), and additional scalp points before the recording.

In order to remove external interference and correct for head motions, the raw MEG data were first pre-processed off-line using the spatio-temporal signal-space separation approach (Taulu and Simola, 2006) implemented in the Maxfilter 2.2 (MEGIN, Helsinki, Finland). The data were then band-pass filtered at 0.1–100 Hz. Cardiac and ocular movement artifacts were removed using ICA algorithm based on EEGLAB toolbox (Delorme and Makeig, 2004) implemented in a custom-made MATLAB code (R2021a, Mathworks Inc., Natick, MA, USA), using ECG and EOG as reference. MEG data were divided into epochs ranging from 1 s before to 3 s after the stimulus onset. Epochs with continuous muscular contraction identified on EMG signal and/or sensor jumps were excluded from further analysis. Finally, data epochs were grouped according to the experimental conditions: hand-related images, hand-related verbs, foot-related images, foot-related verbs. Movement onset was determined by manually tagging the onset of the EMG burst identified as the time point in which the EMG signal exceeded 30% of the maximal voluntary contraction.

### MEG data analysis

Cortical source activations and time-series were estimated using Brainstorm software (Hari et al., 1998). A template brain MRI (MNI/ICBM152, 56), co-registered on MEG data by means of digitized scalp points, was used to generate a realistically shaped single-shell model as volume conductor (BEM model as implemented in OpenMEEG, 57) and ~15,000 dipoles distributed on the brain cortex were defined as source model. The dynamic statistical parametric mapping (dSPM) method (Nishitani, 2000) was employed for the estimation of brain activity at the source level. The noise covariance matrix was calculated using pre-stimulus baseline period data (−1,000–0 ms).

As the inferences were made at the group level, individual source maps were spatially smoothed with a Gaussian kernel with a Full-Width Half Maximum of 3 mm and were averaged for all conditions. Brain sources were grouped according to Destrieux’s atlas (Strafella, 2000) for further analysis.

To explore the language-motor interference effect, we chose the post-stimulus range of 150–350 ms as the period of interest. We selected this period since it has been proposed that concrete linguistic materials are semantically processed in the motor and premotor cortices within 150–170 ms after the stimulus onset (Pulvermüller et al., 2001; Pulvermüller and Shtyrov, 2005; Pulvermüller et al., 2005). Following the experimental hypothesis that understanding semantic of actions recruit brain regions also involved in the execution of those same actions, we selected as the region of interest (ROI) the precentral gyrus (preCG), within the period of interest for all conditions.

The source time series corresponding to each epoch (−2–2.5 s) was extracted from all vertices belonging to the ROI and PCA was used to obtain a single time series for each condition for all the successive comparisons (virtual channel). We used PCA to find the most representative signal in the activated areas since the selected ROI was quite large. Time–frequency representations of virtual channel epochs were computed across frequencies from 1 to 30 Hz (in 1 Hz steps) and time from −2 to 2.5 s (in 0.1 s steps) with a fixed frequency smoothing of 4 Hz by means of multitapers approach. The relative power change course band as compared to the mean of the baseline period was calculated for each epoch and each frequency in the β band (13–30 Hz) by applying the formula [(Epoch(t) − Baseline)/Baseline], where t indicates the time point, then averaged across frequencies and finally averaged for each condition separately. Finally, the Area under Curve (AuC) in the period of interest (150–350 ms) was calculated. Analyses were performed by means of custom Matlab (MATLAB 2021a, MathWorks, Inc., Natick, MA, USA) using functions from the Fieldtrip toolbox (Glenberg and Kaschak, 2002).

### Statistical analysis

RTs and AuC of Beta rhythm were first checked for normality using the Shapiro-Wilk test. Both RTs and AuC of Beta rhythm were compared using repeated measures ANOVA (rmANOVA) with Effector (hand, foot) and Stimulus type (images, verbs) as within participants factors. *Post-hoc* test for stimulus type was performed by means of paired *t*-tests. The significance level was set to 0.05, and values are expressed as mean ± standard deviation.

## Results

### Reaction times

Two participants were excluded from the analysis due to technical problems with MEG signals (presence of sensors’ jumps or epochs with artifacts). The remaining participants performed well, and the overall mean error rate was 2.4%. In particular, the errors of commission (response to a scrambled image or pseudo-verb) were 1.8%, while the errors of omission (non-response to words and pictures depicting hand- or foot-related action) were only 0.5%. Given that the maximum error of omission was one for six subjects (one foot picture, two foot verbs, one hand picture and two hand verbs), all correct answers were analyzed. Shapiro-Wilk test indicates that data were normally distributed (see Table 1). rmANOVA showed main effects of Effector (*F*_(1,12)_ = 24.52, *p* < 0.001) and Stimulus type (*F*_(1,12)_ = 8.89, *p* = 0.011). Inspection of RTs revealed that participants gave slower responses to hand-related actions as compared to foot-related actions, regardless of the presentation modality (images or words). Furthermore, responses to visually presented actions were faster than responses to verbs (see Table 1).

**Table 1:** Descriptive statistic of RTs analysis.

	**Verbs**		**Images**		
	**Mean and Std Dev. (ms)**	**Shapiro-Wilk test (p)**	**Mean and Std Dev. (ms)**	**Shapiro-Wilk test (p)**	**Mean (Effector)**
Foot-related action	489 ± 71.81	0.52	471 ± 61.95	0.91	480 ± 66.40
Hand-related action	540 ± 64.04	0.68	523 ± 46.57	0.19	532 ± 55.55
**Mean (Stimulus Type)**	515 ± 71.50		497 ± 59.98		

Note. Data was normality distributed if the *p*-value of Shapiro-Wilk test was assessed > 0.05.

### MEG data

**Figure 2:**
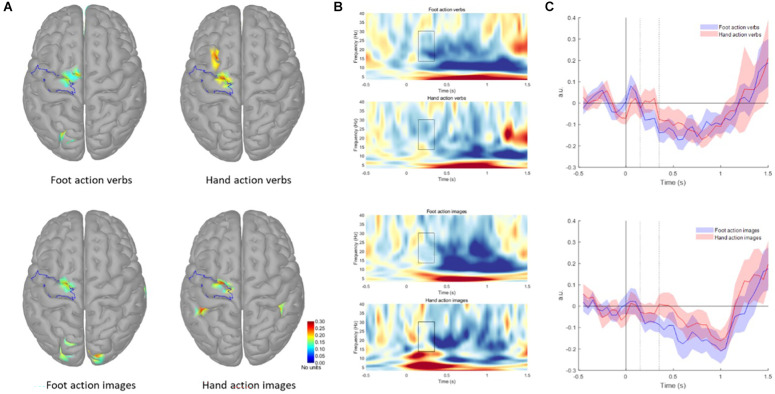
**(A)** Grand average maps of responses to different categories of stimuli in the 150–350 ms period. The highlighted area represents the ROI used for the analysis. The color scale is the same for each map (0–0.3); for illustrative purposes, maps were thresholded at 50% of the maximum amplitude. **(B)** Time-frequency representation of the virtual channel obtained from the ROI for each condition. The highlighted area represents the time and frequency interval selected for the analysis. The color scale is the same for each image (−0.3–0.4). **(C)** Beta band modulation with respect to baseline (−1–0 s) for verbs (upper graph) and images (lower graph) stimuli. Shaded areas indicate the standard error of the mean. Dotted lines indicate the period for AuC calculation, gray dotted line refers to the go signal.

We extracted and analyzed ROI from the pre CG, including the premotor and motor cortex (see Figure 2A). In Figure 2B the time frequency representation of the virtual channel obtained from the ROI for each condition is shown. Shapiro-Wilk test indicates that all AuC values were normally distributed. rmANOVA showed main effect of Effector (*F*_(1, 12)_ = 9.169, *p* = 0.011). In general, the AuC was greater for the foot-related action stimuli as compared to hand-related action ones (Foot-related verbs: −0.57 ± 0.51; Hand-related verbs: −0.10 ± 0.77; Foot-related images: −0.45 ± 0.53; Hand-related images −0.10 ± 0.64). The paired *t*-tests confirmed the greater AuC for Foot-related stimuli in comparison to Hand-related stimuli, regardless of the presentation modalities [Verbs: *t*_(12)_ = −2.31, *p* = 0.039); Images (*t*_(12)_ = −2.47, *p* = 0.030) in pre CG (see Figure 2C)]. For the activation of areas different from pre CG see Supplementary Materials.

## Discussion

The results of the present study showed a slowing down of hand responses to pictures showing hand-related actions, and to hand-related verbs, as compared to pictures and verbs describing foot-related actions, thus confirming the results of a previous behavioral study of our group (Garofalo et al., 2022). In addition, the analysis of the MEG signals provided the neurophysiological correlates of this effect by showing a modulation of Beta rhythm within the pre-central gyrus, coherent with the behavioral results.

Beta rhythm, as revealed by MEG, had a weaker decrease during the processing of hand-related actions, whatever the modality of presentation, as compared to foot-related actions. The so-called ERD, elicited by suppression of Beta rhythm, recorded in pre CG, occurs when motor areas are involved in the actual execution of an action or, to a less degree, when people watch or imagine performing an action (Hari et al., 1998). Our results are in keeping with pivotal studies, showing that this ERD also occurs, although to a less degree, not only during the observation of hand actions but also during the processing of hand-related verbs (Klepp et al., 2015). The presence of ERD for both visually and verbally presented stimuli supports the notion that the same mechanisms and neural structures are working when participants had to give meaning to actions (i.e., semantic processing), whatever the modality of presentation. These findings imply that the brain areas involved in the execution of actions are also recruited during the semantic processing of those actions. Hence, they support further the embodied approach to language processing, showing that the re-enactment of motor structures where actions are represented are crucial to attribute meanings to action words.

One could argue that these findings go counter to some important research that showed facilitation of motor activity during action observation (Fadiga et al., 1995; Cochin, 1999; Nishitani, 2000; Strafella, 2000). When actions are expressed by verbal labels (i.e., verbs), it has been forwarded that a double-stage processing occurs. The first stage occurs very early after stimulus presentation, and it seems to be crucial for understanding. This early processing results from a behavioral point of view in the slowing down of motor responses (Buccino et al., 2005; Boulenger et al., 2006; Sato et al., 2008; Dalla Volta et al., 2009; de Vega et al., 2013; Marino et al., 2014) and from a neurophysiological point of view in a reduction of MEPs amplitude (as revealed by TMS), and a weaker decrease of ERD as showed by MEG (Buccino et al., 2005; Klepp et al., 2015). The second stage is late and occurs when semantic processing has already been completed. In this situation, participants give faster responses (for example action-sentence compatibility effect, ACE; see Del Maschio et al., 2021) or show facilitation of different neurophysiological parameters elicited with different neurophysiological techniques (Watkins and Strafella, 2003; Chersi et al., 2010; de Vega et al., 2013; Klepp et al., 2017, 2019). Considering that, as revealed also by the present results, a substantial motor equivalence exists between observed actions and verbally described actions (Buccino et al., 2016; Hardwick et al., 2018; Garofalo et al., 2022), one may argue that the time course of motor activity during action observation overlaps the one found during the processing of verbally described actions. In detail, when participants engage in a hand motor response, as in our task, during the processing of a seen hand movement, there may be a cost at an early stage. However, when the motor task is completed after the observed action has been fully comprehended, there may be action facilitation. Note that for abstract actions, there may still be a substantial equivalence between sensorimotor experience and verbal description of actions, if it is assumed that they are different from concrete ones because they are anchored in more sophisticated sensory, motor, and emotional experiences than concrete actions (Buccino et al., 2019; Del Maschio et al., 2021) rather than because they are disentangled from experiences or learned through social interactions (Borghi et al., 2013). The complexity of abstract concepts can be defined by the number of biological effectors that can be involved in an abstract action; in the recruitment of different systems (sensory, motor, and emotional); and in the dynamic changes that an abstract action can undergo over time and across cultures, and hence in the different neural substrates subserving those dynamic changes (Buccino et al., 2019).

It is worth stressing that similar recruitment of the motor system may also occur during the processing of visually presented graspable objects and their corresponding nouns (Shinkareva et al., 2011; Gough et al., 2012; Devereux et al., 2013; Marino et al., 2013, 2014; Visani et al., 2022). Also for graspable objects, the neural structures where their motor properties are represented are also re-enacted during the processing of corresponding verbal labels, further supporting the notion of shared semantics.

Despite the fact that there are no single neuron studies supporting the presence of mirror neurons in the humans’ pre-motor and parietal cortices, it is most likely that attributing meaning to verbs has its neuronal counterpart in these neurons (Kemmerer and Gonzalez-Castillo, 2010; Buccino et al., 2016). In a similar vein, one may argue that nouns may have their neuronal counterpart in canonical neurons (Marino et al., 2014; Horoufchin et al., 2018). These neurons, in the monkey, discharge during the grasping of objects and also during the mere observation of those objects (Murata et al., 2000). Accordingly, evidence in humans indicate that during action observation the intrinsic characteristics of the to-be-grasped objects modulate corticospinal excitability and responses to the time-to-contact (Craighero et al., 2014).

It is interesting to observe that when combining verbs and nouns to construct sentences, people choose one precise approach to perform an action from a list of possible ones. For instance, when we say “I hold,” I express all possible ways to hold an object in my hands. However, if a word is added to the sentence, as in “I hold a cup,” then only one particular manner of grasping is re-enacted (Marino et al., 2012). By doing this, we restrict the potential actions and re-enact the action using the best suitable motor representation. In other words, the way a biological effector (such as a hand or foot) interacts with an object in the environment is reflected in the way we construct concrete sentences to depict what occurs in a particular context. Further evidence suggests that adverbs of place (*far* vs. *near*) are also rooted in the sensorimotor system since they are implicitly associated with functionally congruent actions (*look at* vs. *grasp*, Craighero and Marini, 2021), as well as adjectives denoting manipulative qualities are associated with the characteristics of the objects expressed by nouns (Gough et al., 2013; Garofalo et al., 2021).

Overall, these findings imply that rather than being based on *a priori* determined syntactic categories (Vigliocco et al., 2011; Buccino et al., 2016), the distinction between word categories may be anchored in the sensorimotor experience. This proposal offers a perspective for an embodied approach also to the way we combine linguistic words (i.e., syntax). In this respect, some authors have proposed that the syntactic representation of words can be rooted in the activation and interaction of specific neuronal populations (Feldman and Narayanan, 2004; Pulvermüller, 2010). We forward that at least for verbs and nouns this speculation most likely refers to mirror and canonical neurons.

Despite the increasing number of empirical evidence suggesting the common neural substrates for semantic processing, including the present findings, it is worth stressing that in the present study some methodological limitations need to be underlined. First, our sample size was rather small, so future studies including a greater number of participants are needed. Second, our analysis focused on the motor/premotor cortex. Since these areas are strictly connected with the parietal cortex, it could be interesting to study the modulation of brain rhythms also within the parietal lobe during the processing of observed and verbally described actions. This in order to assess whether the fronto-parietal network, known to be involved in action observation and understanding, is also involved in language processing.

## Data Availability Statement

The raw data supporting the conclusions of this article will be made available by the authors, without undue reservation.

## Ethics Statement

The studies involving human participants were reviewed and approved by Ethical Committee of Istituto Neurologico Carlo Besta. The patients/participants provided their written informed consent to participate in this study.

## Author Contributions

EV: resources, data curation, formal analysis, methodology, and writing—original draft. GG: methodology, visualization, formal analysis, and writing—original draft. DR: project administration, methodology, resources, and writing—original draft. DD: data curation and investigation. LC and LR: writing—original draft, and writing—review and editing. GB: conceptualization, methodology, supervision, writing—original draft, and writing—review and editing. All authors contributed to the article and approved the submitted version.
