# Supplementary material for: Grasping the semantic of actions: a combined behavioral and MEG study
Source: Front Hum Neurosci. 2022 Dec 13;16:1008995. doi: 10.3389/fnhum.2022.1008995 (PMC9792482; doi:10.3389/fnhum.2022.1008995)

Supplementary figure.

Mean activation maps at different time-intervals from 80 ms to 350 ms, for each condition. All the maps have the same color scale.


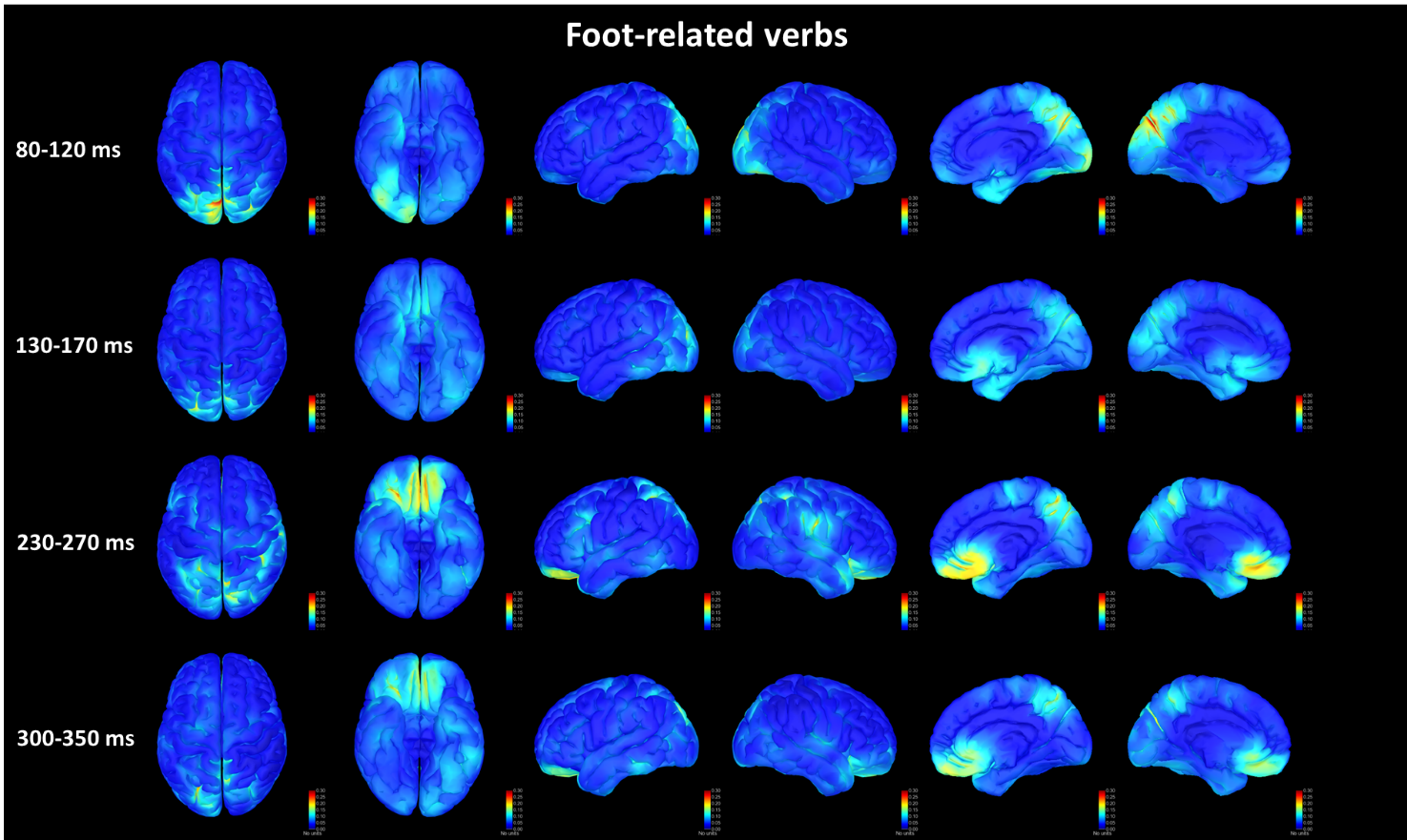


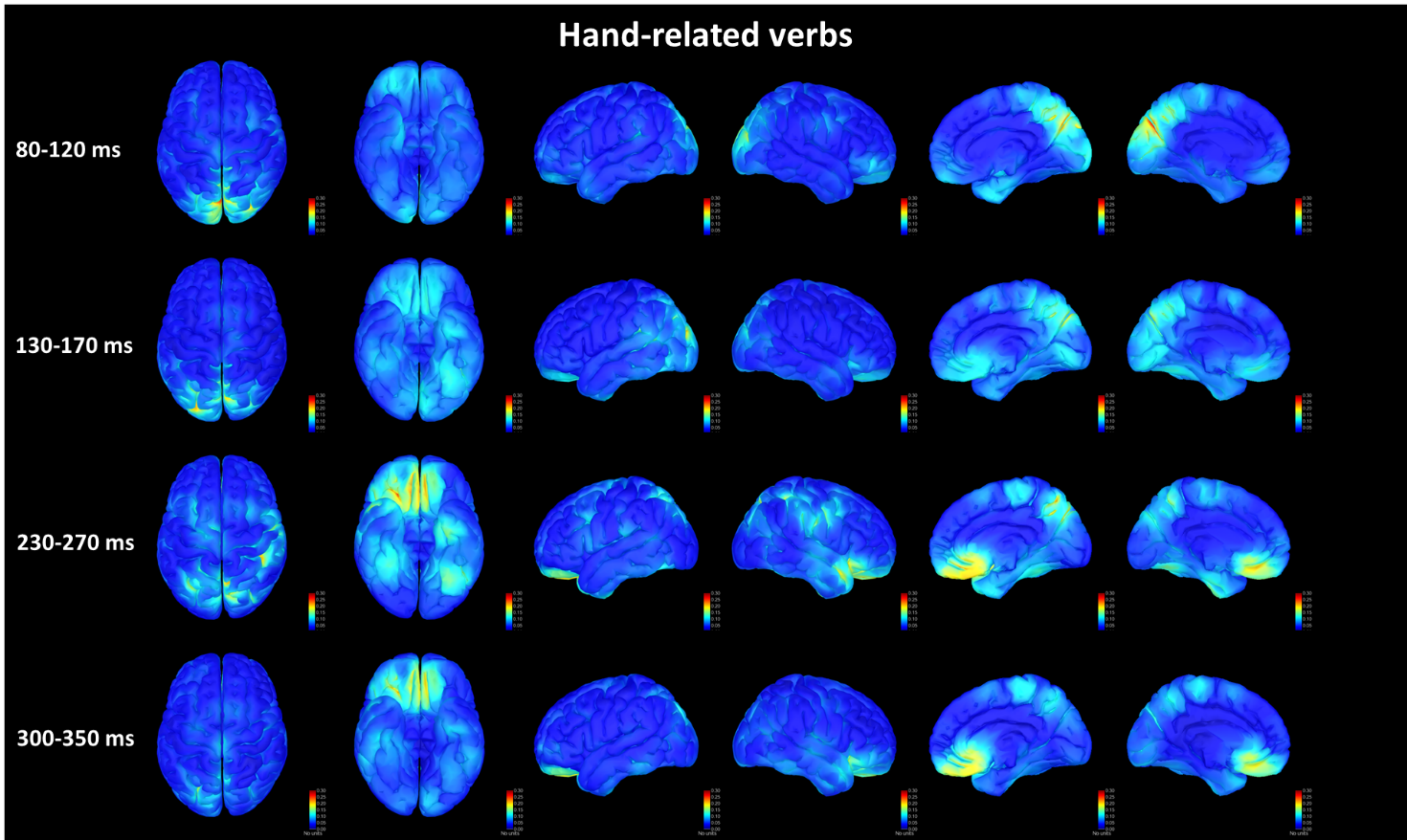


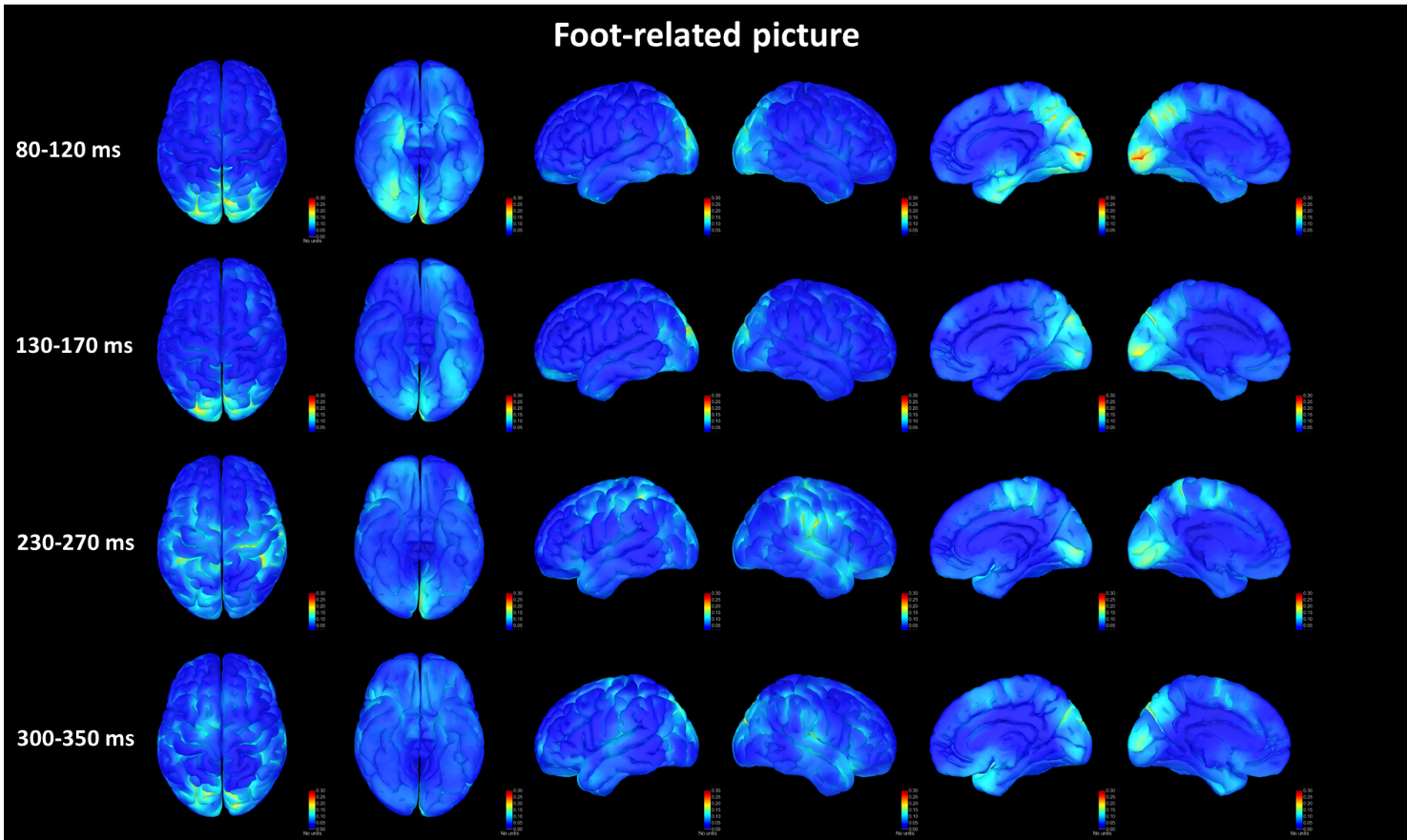


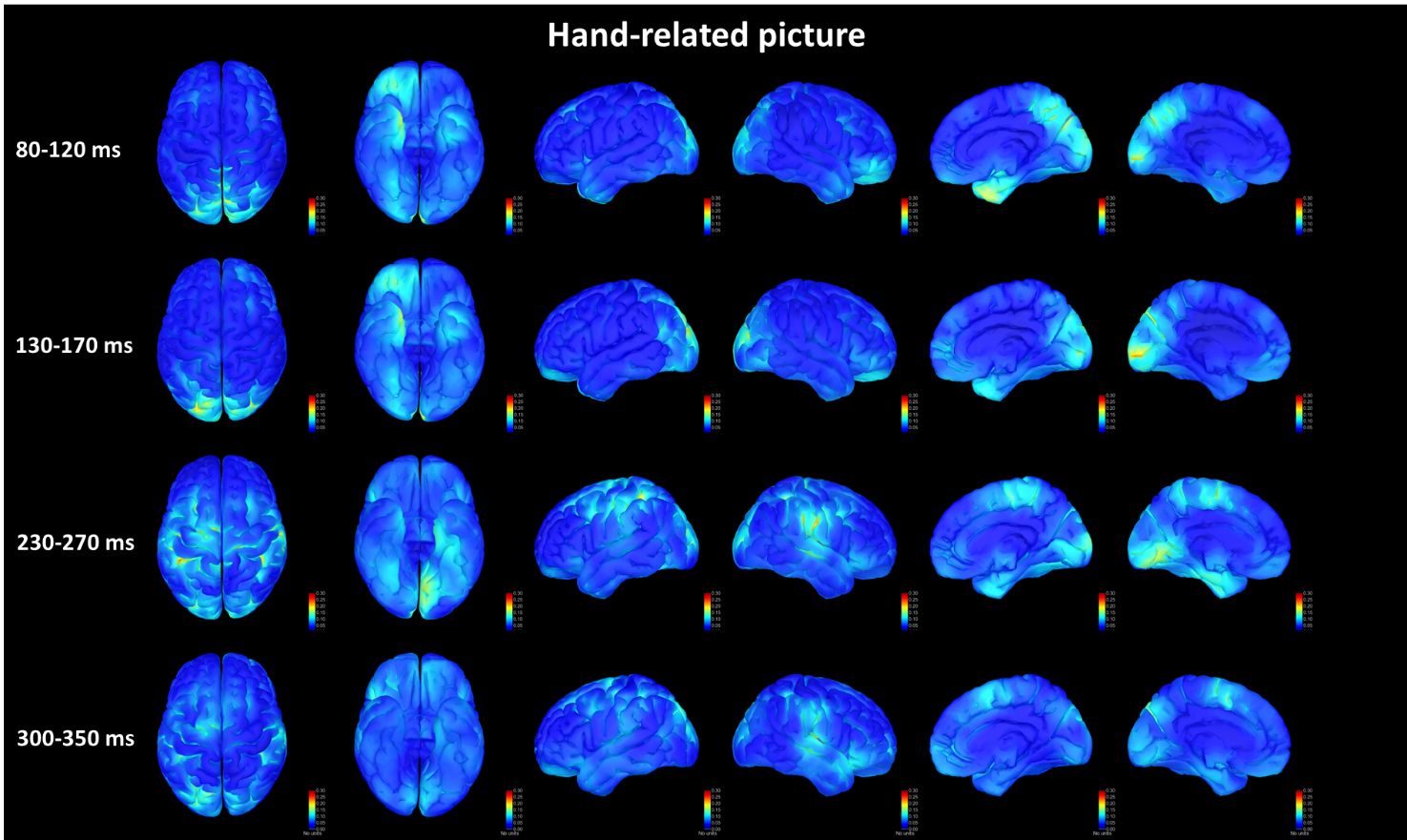

Supplement: Supplementary file 1 [file Data_Sheet_1.docx]
